# Bis[1,2-bis­(meth­oxy­carbon­yl)ethene-1,2-dithiol­ato-κ^2^ *S*,*S*′]bis­(η^5^-penta­methyl­cyclo­penta­dien­yl)tetra-μ_3_-sulfido-tetra­iron(4 *Fe*–*Fe*) hexa­fluoridophosphate

**DOI:** 10.1107/S1600536813007514

**Published:** 2013-03-23

**Authors:** Shinji Inomata, Shohei Ito, Tsugiko Takase

**Affiliations:** a Faculty of Symbiotic Systems Science, Fukushima University, 1 Kanayagawa, Fukushima 960-1296, Japan; b Center for Practical and Project-Based Learning, Cluster of Science and Technology, Fukushima University, 1 Kanayagawa, Fukushima 960-1296, Japan

## Abstract

The asymmetric unit of the title compound, [Fe_4_(C_6_H_6_O_4_S_2_)_2_(C_10_H_15_)_2_S_4_]PF_6_, contains two different complex cations and two PF_6_^−^ anions. The two complex cations have similar conformations with the butterfly-like Fe_4_S_4_ core surrounded by two penta­methyl­cyclo­penta­dienyl ligands and the S atoms of two dithiol­ate ligands. In each Fe_4_S_4_ core, there are four short Fe—Fe and two long Fe⋯Fe contacts, suggesting bonding and non-bonding inter­actions, respectively. The Fe—S distances range from 2.1287 (13) to 2.2706 (16) Å for one and from 2.1233 (13) to 2.2650 (16) Å for the other Fe_4_S_4_ core. The Fe—S distances involving the dithiol­ate ligands are in a more narrow range [2.1764 (16)–2.1874 (13) Å for one and 2.1743 (14)–2.1779 (16) Å for the other cation]. There are no significant inter­actions between cations and anions.

## Related literature

For background to polynuclear transition metal clusters, see: Geiger & Connelly (1985 ▶). For structural details of iron–sulfur cubane-type clusters, see: Blonk *et al.* (1992 ▶); Inomata *et al.* (1994 ▶, 1995 ▶); Schunn *et al.* (1966 ▶); Toan *et al.* (1977*a* ▶,*b* ▶); Wei *et al.* (1966 ▶).
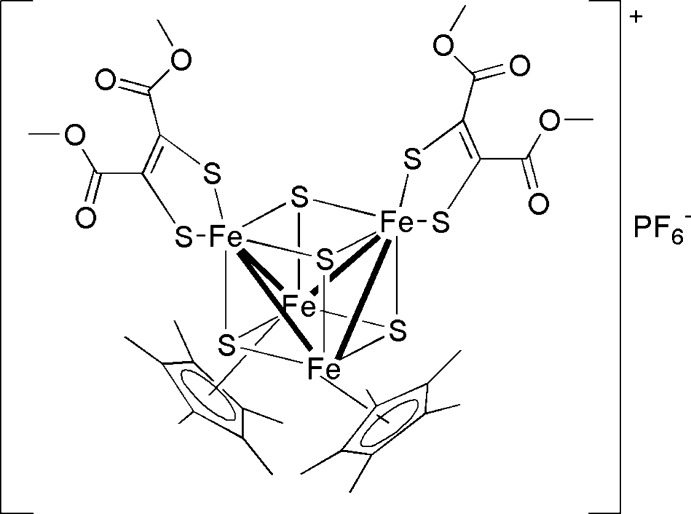


## Experimental

### 

#### Crystal data

[Fe_4_(C_6_H_6_O_4_S_2_)_2_(C_10_H_15_)_2_S_4_]PF_6_
*M* *_r_* = 1179.51
Monoclinic, 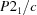

*a* = 15.546 (9) Å
*b* = 23.767 (13) Å
*c* = 24.872 (14) Å
β = 102.209 (6)°
*V* = 8982 (9) Å^3^
*Z* = 8
Mo *K*α radiation
μ = 1.74 mm^−1^
*T* = 93 K
0.20 × 0.20 × 0.15 mm

#### Data collection

Rigaku Saturn diffractometer
Absorption correction: multi-scan (*REQAB*; Jacobson, 1998 ▶) *T*_min_ = 0.634, *T*_max_ = 0.770
81617 measured reflections
20169 independent reflections
16442 reflections with *I* > 2σ(*I*)
*R*_int_ = 0.062

#### Refinement

*R*[*F* ^2^ > 2σ(*F* ^2^)] = 0.075
*wR*(*F* ^2^) = 0.186
*S* = 1.14
20169 reflections
1064 parameters
H-atom parameters constrained
Δρ_max_ = 0.94 e Å^−3^
Δρ_min_ = −0.87 e Å^−3^

### 

Data collection: *CrystalClear* (Rigaku, 2009 ▶); cell refinement: *CrystalClear*; data reduction: *CrystalClear*; program(s) used to solve structure: *SIR97* (Altomare *et al.*, 1999 ▶); program(s) used to refine structure: *SHELXL97* (Sheldrick, 2008 ▶); molecular graphics: *ORTEP-3 for Windows* (Farrugia, 2012 ▶); software used to prepare material for publication: *CrystalStructure* (Rigaku, 2006 ▶).

## Supplementary Material

Crystal structure: contains datablock(s) global, I. DOI: 10.1107/S1600536813007514/wm2732sup1.cif

Structure factors: contains datablock(s) I. DOI: 10.1107/S1600536813007514/wm2732Isup2.hkl

Additional supplementary materials:  crystallographic information; 3D view; checkCIF report

## Figures and Tables

**Table 1:** Selected bond lengths (Å)

Fe1—Fe2	3.1989 (10)
Fe1—Fe3	2.7418 (10)
Fe1—Fe4	2.7306 (10)
Fe2—Fe3	2.7505 (9)
Fe2—Fe4	2.7601 (11)
Fe3—Fe4	3.2499 (11)
Fe5—Fe6	3.1960 (10)
Fe5—Fe7	2.7603 (9)
Fe5—Fe8	2.7619 (11)
Fe6—Fe7	2.7601 (10)
Fe6—Fe8	2.7223 (10)
Fe7—Fe8	3.2216 (11)

**Fig. 1.**
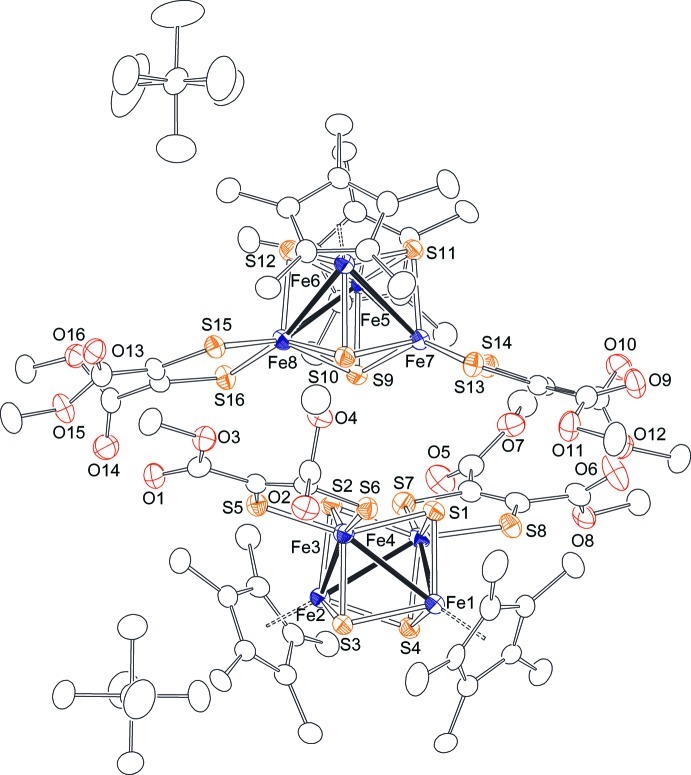
The molecular structures of the two complex cations and the PF6- anions in the title compound, drawn with displacement ellipsoid at the 50% probability level. All hydrogen atoms were omitted for clarity. Solid lines indicate short Fe–Fe contacts.

## supplementary crystallographic information

### Comment

The structures of polynuclear transition metal clusters depend on the total charge of the clusters (Geiger & Connelly, 1985). Notable examples are illustrated by a series of cyclopentadienyl (Cp)-supported iron-sulfur cubane-type clusters, [Fe_4_S_4_Cp_4_] (Schunn *et al.*, 1966; Wei *et al.*, 1966) and their monocationic and dicationic representatives [Fe_4_S_4_Cp_4_]^+^ (Toan *et al.*, 1977*a*) and [Fe_4_S_4_Cp_4_]^2+^ (Toan *et al.*, 1977*b*). In this series, the total bond order of iron—iron interactions increases upon changing the charge from 0 to +2. A similar tendency is also known for methylcyclopentadienyl analogues [Fe_4_S_4_(MeCp)_4_]*^n^* (*n* = 0, +1) (Blonk *et al.*, 1992).

Previously, we found a structural change accompanying an one-electron transfer between the mixed-ligand iron-sulfur cubane-type cluster [Fe_4_S_4_(Ph_2_C_2_S_2_)_2_(C_5_Me_5_)_2_] ([Fe_4_S_4_(Ph_2_C_2_S_2_)_2_Cp*_2_]) and its monocationic derivative [Fe_4_S_4_(Ph_2_C_2_S_2_)_2_Cp*_2_](PF_6_) (Inomata *et al.*, 1994). In both clusters the Fe_4_S_4_ cores are surrounded by two pentamethylcyclopentadienyl (Cp*) ligands and the S atoms of two diphenyldithiolate ligands. Upon single-electron oxidation, the number of iron—iron bonds increased by three to four accompanied by a complex rearrangement of corresponding bonds. We report here the second structural example of such a monocationic mixed-ligand cluster, [Fe_4_S_4_{(MeO_2_C)_2_C_2_S_2_}_2_Cp*_2_]PF_6_ or [Fe_4_S_4_(C_6_H_6_O_4_S_2_)_2_(C_10_H_15_)_2_]PF_6_, (I), which has bis(methoxycarbonyl)dithiolate ligands instead of diphenyldithiolate ligands.

The asymmetric unit of compound (I) consists of two different cations and anions. There are no significant cation···anion interactions. The cations have quite similar conformations. The butterfly-like Fe_4_S_4_ cores are surrounded by two Cp* ligands and the S atoms of two dithiolate ligands. In each Fe_4_S_4_ core, there are four short Fe—Fe contacts (2.7306 (10) - 2.7601 (11) Å and 2.7223 (10) - 2.7619 (11) Å for each cation), indicating there is a bonding interaction. The remaining two long Fe···Fe distances (3.1989 (10) and 3.2499 (11) Å, 3.1960 (10) and 3.2216 (11) Å for each cation) suggest non-bonding interactions (Table 1). The iron-sulfur distances in the Fe_4_S_4_ core range from 2.1287 (13) to 2.2706 (16) Å for one and from 2.1233 (13) to 2.2650 (16) Å for the other cation and are normal. On the other hand, the distances between iron and sulfur involving the dithiolate ligands are in a more narrow range (2.1764 (16) to 2.1874 (13) Å and 2.1743 (14) to 2.1779 (16) Å for each cation). The slight differences in both complex cations are registered by changes of the conformation between methoxycarbonyl groups and dithiolate five-membered chelate rings.

### Experimental

To a dichloromethane solution containing [Fe_4_S_4_{(MeO_2_C)_2_C_2_S_2_}_2_Cp*_2_] (Inomata *et al.*, 1995) (104 mg, 0.101 mmol), was added [Fe(Cp)_2_]PF_6_ (107 mg, 0.323 mmol). The reaction mixture was stirred for 0.5 h at room temperature. After removal of the solvent, the residue was washed with water and then hexane to afford the title compound (yield 109 mg; 92%). Single crystals suitable for X-ray structural analysis were grown by layering hexane on the dichloromethane solution of the monocationic cluster at room temperature.

### Refinement

All hydrogen atoms were placed in calculated positions with C—H distances of 0.98 Å. The *U*_iso_(H) values were fixed at 1.2 times the *U*_eq_(C) values of the carbon atoms to which they are covalently bonded.

### Crystal data

**Table:**

[Fe_4_(C_6_H_6_O_4_S_2_)_2_(C_10_H_15_)_2_S_4_]PF_6_	*F*(000) = 4792.00
*M**_r_* = 1179.51	*D*_x_ = 1.744 Mg m^−^^3^
Monoclinic, *P*2_1_/*c*	Mo *K*α radiation, λ = 0.71075 Å
Hall symbol: -P 2ybc	Cell parameters from 16701 reflections
*a* = 15.546 (9) Å	θ = 3.0–27.5°
*b* = 23.767 (13) Å	µ = 1.74 mm^−^^1^
*c* = 24.872 (14) Å	*T* = 93 K
β = 102.209 (6)°	Block, black
*V* = 8982 (9) Å^3^	0.20 × 0.20 × 0.15 mm
*Z* = 8	

### Data collection

**Table:**

Rigaku Saturn diffractometer	16442 reflections with *I* > 2σ(*I*)
Detector resolution: 7.31 pixels mm^-1^	*R*_int_ = 0.062
ω scans	θ_max_ = 27.5°
Absorption correction: multi-scan (*REQAB*; Jacobson, 1998)	*h* = −20→20
*T*_min_ = 0.634, *T*_max_ = 0.770	*k* = −30→30
81617 measured reflections	*l* = −32→31
20169 independent reflections	

### Refinement

**Table:**

Refinement on *F*^2^	H-atom parameters constrained
*R*[*F*^2^ > 2σ(*F*^2^)] = 0.075	*w* = 1/[σ^2^(*F*_o_^2^) + (0.0697*P*)^2^ + 28.5393*P*] where *P* = (*F*_o_^2^ + 2*F*_c_^2^)/3
*wR*(*F*^2^) = 0.186	(Δ/σ)_max_ < 0.001
*S* = 1.14	Δρ_max_ = 0.94 e Å^−^^3^
20169 reflections	Δρ_min_ = −0.87 e Å^−^^3^
1064 parameters	

### Special details

**Table:**

Refinement. Refinement was performed using all reflections. The weighted *R*-factor (*wR*) and goodness of fit (*S*) are based on *F*^2^. *R*-factor (gt) are based on *F*. The threshold expression of *F*^2^ > 2.0 σ(*F*^2^) is used only for calculating *R*-factor (gt).

### Fractional atomic coordinates and isotropic or equivalent isotropic displacement parameters (Å^2^)

**Table:**

	*x*	*y*	*z*	*U*_iso_*/*U*_eq_	
Fe1	0.07715 (5)	0.25810 (3)	0.13972 (3)	0.02379 (16)	
Fe2	0.17209 (5)	0.14168 (3)	0.12396 (3)	0.02310 (15)	
Fe3	0.25495 (5)	0.23885 (3)	0.16724 (3)	0.02406 (16)	
Fe4	0.10372 (5)	0.17163 (3)	0.21367 (3)	0.02498 (16)	
Fe5	0.40860 (5)	0.16445 (3)	0.41442 (3)	0.02296 (15)	
Fe6	0.51419 (5)	0.27648 (3)	0.39853 (3)	0.02344 (15)	
Fe7	0.33413 (5)	0.26306 (3)	0.36759 (3)	0.02417 (16)	
Fe8	0.48134 (5)	0.19003 (3)	0.32525 (3)	0.02368 (15)	
S1	0.16053 (9)	0.25894 (5)	0.22228 (5)	0.0252 (2)	
S2	0.24502 (9)	0.15484 (5)	0.20734 (5)	0.0249 (2)	
S3	0.16987 (9)	0.22812 (5)	0.08839 (5)	0.0241 (2)	
S4	0.03634 (9)	0.16770 (5)	0.12982 (5)	0.0252 (2)	
S5	0.38789 (9)	0.21638 (5)	0.16024 (5)	0.0267 (2)	
S6	0.29811 (9)	0.32650 (5)	0.17785 (6)	0.0271 (2)	
S7	0.10692 (9)	0.09201 (6)	0.25727 (6)	0.0296 (2)	
S8	0.00484 (10)	0.20097 (6)	0.25752 (6)	0.0304 (3)	
S9	0.33870 (9)	0.17746 (5)	0.32996 (5)	0.0249 (2)	
S10	0.43360 (9)	0.27930 (5)	0.31515 (5)	0.0253 (2)	
S11	0.41556 (9)	0.25207 (5)	0.44758 (5)	0.0247 (2)	
S12	0.54640 (9)	0.18524 (5)	0.40926 (5)	0.0245 (2)	
S13	0.28944 (9)	0.34880 (5)	0.34754 (6)	0.0284 (2)	
S14	0.20265 (9)	0.24286 (6)	0.37925 (6)	0.0276 (2)	
S15	0.58474 (9)	0.21416 (5)	0.28298 (5)	0.0262 (2)	
S16	0.47793 (9)	0.10758 (5)	0.28698 (6)	0.0278 (2)	
P1	0.22590 (10)	−0.06091 (6)	0.00440 (7)	0.0342 (3)	
P2	0.79528 (11)	0.08690 (7)	0.53460 (7)	0.0398 (3)	
F1	0.2900 (2)	−0.05306 (19)	0.06399 (17)	0.0594 (11)	
F2	0.1630 (2)	−0.06931 (19)	−0.05418 (16)	0.0564 (10)	
F3	0.1453 (2)	−0.03742 (17)	0.02790 (17)	0.0511 (9)	
F4	0.2500 (2)	0.00156 (17)	−0.0111 (2)	0.0635 (11)	
F5	0.3070 (2)	−0.08523 (17)	−0.01850 (16)	0.0485 (9)	
F6	0.2040 (2)	−0.12284 (16)	0.02164 (19)	0.0586 (11)	
F7	0.8525 (4)	0.0881 (2)	0.5951 (2)	0.105 (2)	
F8	0.7378 (3)	0.0907 (2)	0.47366 (19)	0.0819 (16)	
F9	0.7742 (3)	0.15229 (19)	0.5407 (2)	0.0773 (14)	
F10	0.8811 (2)	0.10435 (19)	0.5135 (2)	0.0659 (12)	
F11	0.8167 (3)	0.0239 (2)	0.5264 (3)	0.129 (3)	
F12	0.7088 (2)	0.0706 (2)	0.5558 (2)	0.0691 (13)	
O1	0.5672 (2)	0.23236 (16)	0.13678 (16)	0.0331 (8)	
O2	0.4382 (2)	0.41343 (16)	0.12956 (16)	0.0352 (9)	
O3	0.5844 (2)	0.32126 (15)	0.16952 (16)	0.0312 (8)	
O4	0.4903 (2)	0.40029 (15)	0.22064 (15)	0.0304 (8)	
O5	0.0196 (3)	−0.00103 (18)	0.31801 (18)	0.0441 (10)	
O6	−0.0984 (3)	0.19614 (19)	0.3522 (2)	0.0504 (12)	
O7	0.0088 (2)	0.06421 (17)	0.38207 (16)	0.0353 (9)	
O8	−0.1459 (2)	0.11024 (16)	0.32115 (15)	0.0320 (8)	
O9	0.0916 (2)	0.42316 (17)	0.38344 (17)	0.0391 (9)	
O10	0.0418 (2)	0.28168 (18)	0.42666 (16)	0.0383 (9)	
O11	0.1559 (2)	0.43978 (17)	0.31230 (18)	0.0380 (9)	
O12	−0.0060 (2)	0.32613 (18)	0.34611 (17)	0.0359 (9)	
O13	0.7394 (2)	0.19747 (16)	0.23000 (16)	0.0330 (8)	
O14	0.5351 (2)	0.02758 (17)	0.19250 (18)	0.0390 (9)	
O15	0.6840 (2)	0.11810 (16)	0.18545 (17)	0.0358 (9)	
O16	0.6576 (2)	0.02846 (15)	0.26018 (15)	0.0304 (8)	
C1	0.0520 (3)	0.3487 (2)	0.1384 (2)	0.0327 (12)	
C2	−0.0131 (3)	0.3207 (2)	0.1621 (2)	0.0290 (11)	
C3	−0.0598 (3)	0.2821 (2)	0.1229 (2)	0.0280 (11)	
C4	−0.0237 (3)	0.2864 (2)	0.0741 (2)	0.0306 (11)	
C5	0.0438 (3)	0.3280 (2)	0.0841 (2)	0.0314 (12)	
C6	0.1101 (4)	0.3954 (2)	0.1658 (3)	0.0461 (16)	
C7	−0.0340 (4)	0.3378 (2)	0.2156 (2)	0.0414 (14)	
C8	−0.1378 (3)	0.2477 (2)	0.1283 (2)	0.0382 (13)	
C9	−0.0609 (4)	0.2575 (2)	0.0209 (2)	0.0392 (14)	
C10	0.0918 (4)	0.3494 (2)	0.0421 (2)	0.0468 (17)	
C11	0.2685 (3)	0.0860 (2)	0.0984 (2)	0.0272 (11)	
C12	0.2112 (3)	0.1098 (2)	0.0519 (2)	0.0262 (10)	
C13	0.1226 (3)	0.0924 (2)	0.0524 (2)	0.0266 (10)	
C14	0.1260 (3)	0.0584 (2)	0.1003 (2)	0.0285 (11)	
C15	0.2151 (3)	0.0543 (2)	0.1283 (2)	0.0283 (11)	
C16	0.3666 (3)	0.0857 (2)	0.1092 (2)	0.0334 (12)	
C17	0.2391 (3)	0.1419 (2)	0.0063 (2)	0.0310 (11)	
C18	0.0435 (3)	0.1038 (2)	0.0084 (2)	0.0325 (12)	
C19	0.0515 (3)	0.0273 (2)	0.1147 (2)	0.0322 (12)	
C20	0.2508 (4)	0.0182 (2)	0.1773 (2)	0.0354 (13)	
C21	0.4461 (3)	0.2784 (2)	0.1631 (2)	0.0246 (10)	
C22	0.4050 (3)	0.3275 (2)	0.1710 (2)	0.0262 (10)	
C23	0.0278 (3)	0.0968 (2)	0.2964 (2)	0.0294 (11)	
C24	−0.0190 (3)	0.1451 (2)	0.2953 (2)	0.0291 (11)	
C25	0.5377 (3)	0.2737 (2)	0.1542 (2)	0.0274 (11)	
C26	0.4456 (3)	0.3849 (2)	0.1704 (2)	0.0283 (11)	
C27	0.0180 (3)	0.0471 (2)	0.3319 (2)	0.0318 (12)	
C28	−0.0912 (4)	0.1541 (2)	0.3268 (2)	0.0324 (12)	
C29	0.6745 (3)	0.3204 (2)	0.1611 (2)	0.0374 (13)	
C30	0.5421 (4)	0.4515 (2)	0.2211 (2)	0.0419 (14)	
C31	−0.0156 (4)	0.0199 (2)	0.4170 (2)	0.0444 (15)	
C32	−0.2087 (4)	0.1119 (2)	0.3572 (2)	0.0383 (13)	
C33	0.3072 (3)	0.1112 (2)	0.4383 (2)	0.0284 (11)	
C34	0.3604 (3)	0.1372 (2)	0.4852 (2)	0.0289 (11)	
C35	0.4495 (3)	0.1203 (2)	0.4891 (2)	0.0277 (11)	
C36	0.4515 (3)	0.0821 (2)	0.4438 (2)	0.0260 (10)	
C37	0.3647 (3)	0.0771 (2)	0.4131 (2)	0.0269 (11)	
C38	0.2090 (3)	0.1103 (2)	0.4216 (2)	0.0337 (12)	
C39	0.3257 (4)	0.1734 (2)	0.5250 (2)	0.0322 (12)	
C40	0.5267 (3)	0.1355 (2)	0.5338 (2)	0.0325 (12)	
C41	0.5300 (4)	0.0489 (2)	0.4351 (2)	0.0339 (12)	
C42	0.3336 (4)	0.0391 (2)	0.3645 (2)	0.0312 (11)	
C43	0.6137 (3)	0.3332 (2)	0.3789 (2)	0.0267 (10)	
C44	0.6534 (3)	0.2943 (2)	0.4195 (2)	0.0274 (11)	
C45	0.6133 (3)	0.3026 (2)	0.4661 (2)	0.0282 (11)	
C46	0.5495 (3)	0.3462 (2)	0.4536 (2)	0.0307 (11)	
C47	0.5483 (3)	0.3649 (2)	0.3989 (2)	0.0276 (11)	
C48	0.6428 (3)	0.3458 (2)	0.3264 (2)	0.0325 (12)	
C49	0.7296 (3)	0.2568 (2)	0.4180 (2)	0.0350 (12)	
C50	0.6428 (4)	0.2744 (2)	0.5207 (2)	0.0360 (13)	
C51	0.4979 (4)	0.3719 (2)	0.4914 (2)	0.0387 (14)	
C52	0.4998 (4)	0.4141 (2)	0.3702 (2)	0.0392 (13)	
C53	0.1828 (3)	0.3524 (2)	0.3569 (2)	0.0259 (10)	
C54	0.1439 (3)	0.3045 (2)	0.3713 (2)	0.0261 (10)	
C55	0.6124 (3)	0.1543 (2)	0.2519 (2)	0.0264 (10)	
C56	0.5647 (3)	0.1066 (2)	0.2538 (2)	0.0262 (10)	
C57	0.1386 (3)	0.4085 (2)	0.3535 (2)	0.0285 (11)	
C58	0.0551 (3)	0.3018 (2)	0.3847 (2)	0.0282 (11)	
C59	0.6862 (3)	0.1601 (2)	0.2221 (2)	0.0297 (11)	
C60	0.5833 (3)	0.0505 (2)	0.2305 (2)	0.0282 (11)	
C61	0.1148 (4)	0.4949 (2)	0.3070 (3)	0.0434 (15)	
C62	−0.0926 (4)	0.3320 (2)	0.3588 (2)	0.0424 (14)	
C63	0.7511 (4)	0.1218 (2)	0.1529 (2)	0.0407 (14)	
C64	0.6837 (4)	−0.0250 (2)	0.2398 (2)	0.0361 (13)	
H1	0.0989	0.4021	0.2026	0.055*	
H2	0.0974	0.4298	0.1437	0.055*	
H3	0.1719	0.3849	0.1688	0.055*	
H4	−0.0824	0.3146	0.2229	0.050*	
H5	−0.0513	0.3775	0.2139	0.050*	
H6	0.0181	0.3324	0.2452	0.050*	
H7	−0.1611	0.2283	0.0935	0.046*	
H8	−0.1833	0.2723	0.1374	0.046*	
H9	−0.1202	0.2198	0.1577	0.046*	
H10	−0.0271	0.2683	−0.0066	0.047*	
H11	−0.1225	0.2686	0.0081	0.047*	
H12	−0.0574	0.2167	0.0263	0.047*	
H13	0.1347	0.3779	0.0588	0.056*	
H14	0.0497	0.3662	0.0114	0.056*	
H15	0.1224	0.3182	0.0285	0.056*	
H16	0.3864	0.1075	0.0806	0.040*	
H17	0.3877	0.0469	0.1089	0.040*	
H18	0.3901	0.1027	0.1453	0.040*	
H19	0.1873	0.1500	−0.0228	0.037*	
H20	0.2810	0.1192	−0.0089	0.037*	
H21	0.2670	0.1773	0.0208	0.037*	
H22	−0.0080	0.0860	0.0181	0.039*	
H23	0.0525	0.0884	−0.0265	0.039*	
H24	0.0340	0.1445	0.0048	0.039*	
H25	0.0723	0.0060	0.1487	0.039*	
H26	0.0269	0.0012	0.0848	0.039*	
H27	0.0059	0.0539	0.1200	0.039*	
H28	0.3150	0.0218	0.1871	0.042*	
H29	0.2350	−0.0212	0.1686	0.042*	
H30	0.2257	0.0303	0.2084	0.042*	
H31	0.7139	0.3059	0.1943	0.045*	
H32	0.6924	0.3587	0.1538	0.045*	
H33	0.6777	0.2962	0.1298	0.045*	
H34	0.6012	0.4417	0.2163	0.050*	
H35	0.5464	0.4709	0.2563	0.050*	
H36	0.5135	0.4762	0.1911	0.050*	
H37	−0.0798	0.0174	0.4108	0.053*	
H38	0.0085	0.0289	0.4557	0.053*	
H39	0.0083	−0.0162	0.4079	0.053*	
H40	−0.1833	0.0930	0.3919	0.046*	
H41	−0.2630	0.0927	0.3393	0.046*	
H42	−0.2219	0.1512	0.3645	0.046*	
H43	0.1841	0.1347	0.4462	0.040*	
H44	0.1878	0.0717	0.4241	0.040*	
H45	0.1908	0.1237	0.3837	0.040*	
H46	0.3742	0.1845	0.5551	0.039*	
H47	0.2817	0.1524	0.5399	0.039*	
H48	0.2984	0.2072	0.5061	0.039*	
H49	0.5795	0.1166	0.5272	0.039*	
H50	0.5156	0.1236	0.5694	0.039*	
H51	0.5356	0.1763	0.5339	0.039*	
H52	0.5131	0.0259	0.4018	0.041*	
H53	0.5507	0.0244	0.4668	0.041*	
H54	0.5772	0.0748	0.4309	0.041*	
H55	0.2697	0.0428	0.3521	0.037*	
H56	0.3483	0.0001	0.3753	0.037*	
H57	0.3625	0.0498	0.3347	0.037*	
H58	0.6906	0.3203	0.3226	0.039*	
H59	0.6636	0.3848	0.3271	0.039*	
H60	0.5932	0.3407	0.2952	0.039*	
H61	0.7476	0.2377	0.4535	0.042*	
H62	0.7787	0.2793	0.4107	0.042*	
H63	0.7125	0.2288	0.3888	0.042*	
H64	0.6080	0.2885	0.5464	0.043*	
H65	0.7052	0.2826	0.5351	0.043*	
H66	0.6346	0.2337	0.5163	0.043*	
H67	0.4604	0.4020	0.4721	0.046*	
H68	0.5382	0.3876	0.5236	0.046*	
H69	0.4610	0.3431	0.5034	0.046*	
H70	0.5156	0.4193	0.3345	0.047*	
H71	0.5155	0.4480	0.3927	0.047*	
H72	0.4363	0.4075	0.3648	0.047*	
H73	0.0568	0.4925	0.2822	0.052*	
H74	0.1518	0.5214	0.2918	0.052*	
H75	0.1081	0.5080	0.3432	0.052*	
H76	−0.1281	0.2987	0.3458	0.051*	
H77	−0.1216	0.3656	0.3404	0.051*	
H78	−0.0866	0.3357	0.3986	0.051*	
H79	0.7283	0.1433	0.1193	0.049*	
H80	0.7670	0.0838	0.1430	0.049*	
H81	0.8033	0.1406	0.1743	0.049*	
H82	0.6572	−0.0560	0.2566	0.043*	
H83	0.7480	−0.0284	0.2493	0.043*	
H84	0.6635	−0.0265	0.1997	0.043*	

### Atomic displacement parameters (Å^2^)

**Table:**

	*U*^11^	*U*^22^	*U*^33^	*U*^12^	*U*^13^	*U*^23^
Fe1	0.0243 (4)	0.0269 (3)	0.0205 (3)	0.0010 (2)	0.0056 (2)	−0.0003 (2)
Fe2	0.0239 (4)	0.0253 (3)	0.0202 (3)	−0.0000 (2)	0.0049 (2)	−0.0006 (2)
Fe3	0.0241 (4)	0.0261 (3)	0.0219 (3)	−0.0013 (2)	0.0047 (2)	−0.0004 (2)
Fe4	0.0274 (4)	0.0279 (3)	0.0203 (3)	−0.0021 (2)	0.0065 (3)	0.0003 (2)
Fe5	0.0243 (4)	0.0259 (3)	0.0190 (3)	−0.0008 (2)	0.0053 (2)	0.0001 (2)
Fe6	0.0236 (4)	0.0250 (3)	0.0219 (3)	0.0002 (2)	0.0050 (2)	0.0004 (2)
Fe7	0.0236 (4)	0.0269 (3)	0.0225 (3)	0.0007 (2)	0.0061 (3)	−0.0003 (2)
Fe8	0.0253 (4)	0.0257 (3)	0.0208 (3)	0.0003 (2)	0.0067 (2)	−0.0003 (2)
S1	0.0284 (7)	0.0277 (6)	0.0200 (6)	−0.0012 (5)	0.0062 (5)	−0.0009 (4)
S2	0.0262 (6)	0.0286 (6)	0.0197 (6)	−0.0003 (5)	0.0039 (4)	0.0012 (4)
S3	0.0254 (6)	0.0259 (6)	0.0214 (6)	0.0004 (4)	0.0060 (4)	−0.0005 (4)
S4	0.0251 (6)	0.0281 (6)	0.0231 (6)	−0.0019 (4)	0.0066 (5)	−0.0021 (4)
S5	0.0265 (7)	0.0262 (6)	0.0283 (6)	−0.0011 (5)	0.0076 (5)	−0.0004 (4)
S6	0.0260 (7)	0.0275 (6)	0.0285 (6)	−0.0006 (5)	0.0074 (5)	−0.0012 (4)
S7	0.0304 (7)	0.0302 (6)	0.0295 (6)	−0.0009 (5)	0.0092 (5)	0.0048 (5)
S8	0.0365 (8)	0.0304 (6)	0.0275 (6)	−0.0014 (5)	0.0138 (5)	0.0014 (5)
S9	0.0251 (6)	0.0283 (6)	0.0211 (6)	−0.0002 (5)	0.0045 (4)	−0.0012 (4)
S10	0.0269 (7)	0.0269 (6)	0.0226 (6)	0.0016 (5)	0.0064 (5)	0.0013 (4)
S11	0.0252 (6)	0.0285 (6)	0.0211 (6)	−0.0012 (4)	0.0066 (5)	−0.0024 (4)
S12	0.0243 (6)	0.0259 (6)	0.0237 (6)	0.0001 (4)	0.0060 (5)	0.0020 (4)
S13	0.0255 (7)	0.0270 (6)	0.0343 (7)	0.0014 (5)	0.0101 (5)	0.0024 (5)
S14	0.0259 (7)	0.0290 (6)	0.0289 (6)	0.0001 (5)	0.0080 (5)	0.0014 (4)
S15	0.0287 (7)	0.0261 (6)	0.0256 (6)	−0.0001 (5)	0.0100 (5)	−0.0002 (4)
S16	0.0296 (7)	0.0264 (6)	0.0300 (6)	−0.0018 (5)	0.0120 (5)	−0.0028 (4)
P1	0.0307 (8)	0.0327 (7)	0.0378 (8)	0.0043 (6)	0.0042 (6)	−0.0039 (6)
P2	0.0305 (8)	0.0413 (8)	0.0466 (9)	0.0042 (6)	0.0063 (7)	0.0020 (6)
F1	0.043 (2)	0.080 (3)	0.048 (2)	0.005 (2)	−0.0043 (18)	−0.019 (2)
F2	0.039 (2)	0.088 (3)	0.037 (2)	0.016 (2)	−0.0025 (17)	−0.0132 (19)
F3	0.040 (2)	0.058 (2)	0.059 (2)	0.0031 (17)	0.0181 (18)	−0.0211 (18)
F4	0.055 (2)	0.044 (2)	0.093 (3)	0.0044 (19)	0.022 (2)	0.012 (2)
F5	0.034 (2)	0.060 (2)	0.052 (2)	0.0122 (17)	0.0097 (17)	−0.0066 (17)
F6	0.059 (2)	0.040 (2)	0.079 (3)	0.0003 (18)	0.020 (2)	0.0072 (19)
F7	0.094 (4)	0.139 (5)	0.062 (3)	−0.019 (3)	−0.027 (3)	0.029 (3)
F8	0.050 (2)	0.146 (5)	0.049 (2)	−0.027 (2)	0.007 (2)	−0.005 (2)
F9	0.069 (3)	0.050 (2)	0.119 (4)	0.015 (2)	0.033 (3)	−0.002 (2)
F10	0.032 (2)	0.070 (2)	0.101 (3)	−0.0088 (19)	0.024 (2)	−0.020 (2)
F11	0.058 (3)	0.038 (2)	0.291 (9)	0.001 (2)	0.038 (4)	−0.015 (3)
F12	0.049 (2)	0.088 (3)	0.074 (3)	0.005 (2)	0.022 (2)	0.039 (2)
O1	0.034 (2)	0.032 (2)	0.035 (2)	0.0043 (16)	0.0100 (17)	−0.0044 (15)
O2	0.043 (2)	0.029 (2)	0.032 (2)	−0.0027 (17)	0.0073 (18)	0.0041 (15)
O3	0.028 (2)	0.0302 (19)	0.036 (2)	−0.0039 (15)	0.0091 (16)	0.0009 (15)
O4	0.035 (2)	0.0291 (19)	0.0271 (19)	−0.0053 (16)	0.0074 (16)	−0.0000 (14)
O5	0.057 (3)	0.037 (2)	0.042 (2)	−0.006 (2)	0.019 (2)	0.0018 (18)
O6	0.068 (3)	0.040 (2)	0.054 (2)	−0.014 (2)	0.039 (2)	−0.017 (2)
O7	0.035 (2)	0.043 (2)	0.029 (2)	−0.0070 (17)	0.0083 (17)	0.0103 (16)
O8	0.033 (2)	0.039 (2)	0.027 (2)	−0.0053 (16)	0.0148 (16)	0.0004 (15)
O9	0.048 (2)	0.039 (2)	0.036 (2)	0.0118 (18)	0.023 (2)	0.0049 (17)
O10	0.040 (2)	0.050 (2)	0.029 (2)	−0.0019 (19)	0.0160 (18)	0.0084 (17)
O11	0.037 (2)	0.038 (2)	0.045 (2)	0.0100 (17)	0.0206 (19)	0.0114 (17)
O12	0.021 (2)	0.055 (2)	0.034 (2)	0.0019 (17)	0.0101 (16)	0.0026 (17)
O13	0.026 (2)	0.036 (2)	0.038 (2)	−0.0012 (16)	0.0097 (17)	0.0024 (16)
O14	0.040 (2)	0.032 (2)	0.042 (2)	0.0044 (17)	0.0013 (19)	−0.0084 (17)
O15	0.046 (2)	0.032 (2)	0.036 (2)	−0.0002 (17)	0.0237 (19)	−0.0039 (16)
O16	0.032 (2)	0.0286 (19)	0.031 (2)	0.0034 (15)	0.0067 (16)	0.0008 (14)
C1	0.034 (3)	0.028 (2)	0.038 (3)	0.010 (2)	0.010 (2)	0.003 (2)
C2	0.030 (3)	0.034 (2)	0.024 (2)	0.011 (2)	0.009 (2)	0.006 (2)
C3	0.023 (2)	0.037 (2)	0.023 (2)	0.009 (2)	0.003 (2)	0.003 (2)
C4	0.028 (3)	0.039 (3)	0.024 (2)	0.011 (2)	0.005 (2)	0.005 (2)
C5	0.025 (2)	0.034 (2)	0.037 (3)	0.015 (2)	0.011 (2)	0.012 (2)
C6	0.036 (3)	0.028 (3)	0.072 (4)	0.004 (2)	0.007 (3)	−0.005 (2)
C7	0.050 (4)	0.048 (3)	0.029 (3)	0.016 (2)	0.014 (2)	−0.001 (2)
C8	0.023 (3)	0.046 (3)	0.046 (3)	0.002 (2)	0.009 (2)	0.004 (2)
C9	0.033 (3)	0.054 (3)	0.026 (2)	0.017 (2)	−0.003 (2)	−0.001 (2)
C10	0.046 (4)	0.050 (3)	0.052 (4)	0.019 (3)	0.027 (3)	0.025 (3)
C11	0.028 (2)	0.026 (2)	0.027 (2)	0.003 (2)	0.006 (2)	−0.0046 (19)
C12	0.028 (2)	0.027 (2)	0.024 (2)	0.000 (2)	0.006 (2)	−0.0036 (19)
C13	0.027 (2)	0.027 (2)	0.026 (2)	−0.005 (2)	0.004 (2)	−0.0065 (19)
C14	0.034 (3)	0.028 (2)	0.025 (2)	−0.003 (2)	0.010 (2)	−0.0072 (19)
C15	0.035 (3)	0.024 (2)	0.025 (2)	0.001 (2)	0.003 (2)	−0.0027 (19)
C16	0.028 (3)	0.033 (2)	0.040 (3)	0.002 (2)	0.008 (2)	−0.006 (2)
C17	0.037 (3)	0.039 (3)	0.020 (2)	0.002 (2)	0.012 (2)	−0.002 (2)
C18	0.031 (3)	0.041 (3)	0.026 (2)	−0.002 (2)	0.005 (2)	−0.003 (2)
C19	0.035 (3)	0.030 (2)	0.034 (3)	−0.008 (2)	0.013 (2)	−0.003 (2)
C20	0.047 (3)	0.025 (2)	0.035 (3)	0.008 (2)	0.009 (2)	0.006 (2)
C21	0.023 (2)	0.032 (2)	0.019 (2)	−0.003 (2)	0.0063 (19)	0.0012 (19)
C22	0.029 (2)	0.030 (2)	0.019 (2)	−0.004 (2)	0.004 (2)	0.0007 (18)
C23	0.027 (2)	0.037 (2)	0.025 (2)	−0.007 (2)	0.007 (2)	0.000 (2)
C24	0.031 (3)	0.035 (2)	0.021 (2)	−0.009 (2)	0.006 (2)	−0.002 (2)
C25	0.026 (2)	0.029 (2)	0.027 (2)	−0.001 (2)	0.003 (2)	0.005 (2)
C26	0.031 (3)	0.026 (2)	0.031 (2)	−0.002 (2)	0.011 (2)	−0.001 (2)
C27	0.026 (3)	0.040 (3)	0.029 (2)	−0.008 (2)	0.006 (2)	0.005 (2)
C28	0.039 (3)	0.035 (2)	0.025 (2)	−0.005 (2)	0.010 (2)	−0.000 (2)
C29	0.023 (3)	0.045 (3)	0.046 (3)	−0.002 (2)	0.012 (2)	0.002 (2)
C30	0.041 (3)	0.031 (3)	0.053 (3)	−0.013 (2)	0.012 (3)	0.000 (2)
C31	0.049 (4)	0.051 (3)	0.035 (3)	−0.001 (3)	0.014 (2)	0.012 (2)
C32	0.035 (3)	0.048 (3)	0.035 (3)	0.001 (2)	0.015 (2)	0.004 (2)
C33	0.023 (2)	0.033 (2)	0.031 (2)	−0.004 (2)	0.008 (2)	0.002 (2)
C34	0.027 (2)	0.040 (3)	0.022 (2)	0.003 (2)	0.009 (2)	0.002 (2)
C35	0.031 (3)	0.030 (2)	0.023 (2)	−0.002 (2)	0.007 (2)	0.0024 (19)
C36	0.030 (2)	0.028 (2)	0.019 (2)	0.001 (2)	0.005 (2)	0.0036 (18)
C37	0.031 (3)	0.026 (2)	0.025 (2)	−0.005 (2)	0.010 (2)	0.0052 (19)
C38	0.032 (3)	0.038 (3)	0.033 (3)	−0.003 (2)	0.010 (2)	0.001 (2)
C39	0.037 (3)	0.043 (3)	0.020 (2)	−0.004 (2)	0.013 (2)	−0.001 (2)
C40	0.025 (2)	0.048 (3)	0.023 (2)	0.001 (2)	0.003 (2)	0.006 (2)
C41	0.040 (3)	0.030 (2)	0.035 (3)	0.005 (2)	0.014 (2)	0.001 (2)
C42	0.038 (3)	0.029 (2)	0.028 (2)	−0.007 (2)	0.009 (2)	−0.003 (2)
C43	0.025 (2)	0.027 (2)	0.030 (2)	−0.005 (2)	0.010 (2)	−0.0025 (19)
C44	0.023 (2)	0.029 (2)	0.029 (2)	−0.006 (2)	0.003 (2)	−0.002 (2)
C45	0.027 (2)	0.033 (2)	0.025 (2)	−0.005 (2)	0.006 (2)	−0.005 (2)
C46	0.028 (3)	0.028 (2)	0.038 (3)	−0.005 (2)	0.010 (2)	−0.008 (2)
C47	0.026 (2)	0.023 (2)	0.033 (2)	−0.005 (2)	0.004 (2)	0.002 (2)
C48	0.032 (3)	0.039 (3)	0.028 (2)	−0.005 (2)	0.008 (2)	0.005 (2)
C49	0.032 (3)	0.033 (3)	0.039 (3)	0.002 (2)	0.006 (2)	−0.000 (2)
C50	0.036 (3)	0.043 (3)	0.027 (2)	−0.003 (2)	0.004 (2)	0.004 (2)
C51	0.037 (3)	0.038 (3)	0.046 (3)	−0.010 (2)	0.019 (2)	−0.019 (2)
C52	0.040 (3)	0.028 (2)	0.048 (3)	0.001 (2)	0.006 (2)	0.006 (2)
C53	0.025 (2)	0.035 (2)	0.019 (2)	0.002 (2)	0.006 (2)	0.0027 (19)
C54	0.030 (2)	0.034 (2)	0.016 (2)	0.001 (2)	0.007 (2)	0.0028 (18)
C55	0.032 (3)	0.027 (2)	0.022 (2)	0.004 (2)	0.010 (2)	−0.0009 (19)
C56	0.028 (2)	0.031 (2)	0.020 (2)	0.006 (2)	0.004 (2)	0.0001 (19)
C57	0.027 (2)	0.029 (2)	0.030 (2)	−0.000 (2)	0.006 (2)	−0.001 (2)
C58	0.028 (2)	0.027 (2)	0.030 (2)	0.000 (2)	0.008 (2)	−0.004 (2)
C59	0.031 (3)	0.029 (2)	0.031 (2)	0.005 (2)	0.011 (2)	0.004 (2)
C60	0.032 (3)	0.023 (2)	0.032 (2)	0.004 (2)	0.013 (2)	0.001 (2)
C61	0.039 (3)	0.037 (3)	0.061 (4)	0.005 (2)	0.025 (3)	0.009 (2)
C62	0.027 (3)	0.053 (3)	0.050 (3)	0.003 (2)	0.013 (2)	−0.000 (2)
C63	0.044 (3)	0.045 (3)	0.041 (3)	−0.000 (2)	0.029 (2)	0.000 (2)
C64	0.041 (3)	0.029 (2)	0.041 (3)	0.008 (2)	0.016 (2)	−0.003 (2)

### Geometric parameters (Å, º)

**Table:**

Fe1—Fe3	2.7418 (10)	C22—C26	1.506 (7)
Fe1—Fe4	2.7306 (10)	C23—C24	1.356 (7)
Fe1—S1	2.1844 (13)	C23—C27	1.501 (8)
Fe1—S3	2.2347 (16)	C24—C28	1.513 (8)
Fe1—S4	2.2390 (13)	C33—C34	1.421 (7)
Fe1—C1	2.187 (5)	C33—C37	1.443 (8)
Fe1—C2	2.195 (5)	C33—C38	1.494 (7)
Fe1—C3	2.159 (5)	C34—C35	1.426 (8)
Fe1—C4	2.119 (5)	C34—C39	1.496 (8)
Fe1—C5	2.154 (5)	C35—C36	1.452 (7)
Fe2—Fe3	2.7505 (9)	C35—C40	1.497 (7)
Fe2—Fe4	2.7601 (11)	C36—C37	1.408 (7)
Fe2—S2	2.1656 (13)	C36—C41	1.506 (8)
Fe2—S3	2.2342 (14)	C37—C42	1.504 (7)
Fe2—S4	2.2333 (16)	C43—C44	1.412 (7)
Fe2—C11	2.193 (5)	C43—C47	1.437 (8)
Fe2—C12	2.148 (5)	C43—C48	1.499 (8)
Fe2—C13	2.134 (4)	C44—C45	1.442 (8)
Fe2—C14	2.145 (5)	C44—C49	1.488 (8)
Fe2—C15	2.178 (5)	C45—C46	1.422 (7)
Fe3—S1	2.2598 (16)	C45—C50	1.496 (7)
Fe3—S2	2.2517 (14)	C46—C47	1.428 (8)
Fe3—S3	2.1363 (13)	C46—C51	1.490 (9)
Fe3—S5	2.1769 (16)	C47—C52	1.490 (7)
Fe3—S6	2.1874 (13)	C53—C54	1.372 (7)
Fe4—S1	2.2476 (14)	C53—C57	1.495 (7)
Fe4—S2	2.2706 (16)	C54—C58	1.488 (8)
Fe4—S4	2.1287 (13)	C55—C56	1.360 (7)
Fe4—S7	2.1764 (16)	C55—C59	1.498 (8)
Fe4—S8	2.1790 (18)	C56—C60	1.507 (7)
Fe5—Fe7	2.7603 (9)	C6—H1	0.980
Fe5—Fe8	2.7619 (11)	C6—H2	0.980
Fe5—S9	2.1735 (13)	C6—H3	0.980
Fe5—S11	2.2340 (13)	C7—H4	0.980
Fe5—S12	2.2285 (16)	C7—H5	0.980
Fe5—C33	2.199 (5)	C7—H6	0.980
Fe5—C34	2.154 (5)	C8—H7	0.980
Fe5—C35	2.112 (5)	C8—H8	0.980
Fe5—C36	2.146 (5)	C8—H9	0.980
Fe5—C37	2.183 (5)	C9—H10	0.980
Fe6—Fe7	2.7601 (10)	C9—H11	0.980
Fe6—Fe8	2.7223 (10)	C9—H12	0.980
Fe6—S10	2.1854 (13)	C10—H13	0.980
Fe6—S11	2.2287 (16)	C10—H14	0.980
Fe6—S12	2.2289 (13)	C10—H15	0.980
Fe6—C43	2.184 (5)	C16—H16	0.980
Fe6—C44	2.159 (5)	C16—H17	0.980
Fe6—C45	2.118 (5)	C16—H18	0.980
Fe6—C46	2.147 (5)	C17—H19	0.980
Fe6—C47	2.166 (5)	C17—H20	0.980
Fe7—S9	2.2468 (14)	C17—H21	0.980
Fe7—S10	2.2571 (16)	C18—H22	0.980
Fe7—S11	2.1363 (13)	C18—H23	0.980
Fe7—S13	2.1765 (13)	C18—H24	0.980
Fe7—S14	2.1779 (16)	C19—H25	0.980
Fe8—S9	2.2650 (16)	C19—H26	0.980
Fe8—S10	2.2442 (14)	C19—H27	0.980
Fe8—S12	2.1233 (13)	C20—H28	0.980
Fe8—S15	2.1755 (16)	C20—H29	0.980
Fe8—S16	2.1743 (14)	C20—H30	0.980
S5—C21	1.724 (5)	C29—H31	0.980
S6—C22	1.706 (5)	C29—H32	0.980
S7—C23	1.727 (6)	C29—H33	0.980
S8—C24	1.712 (5)	C30—H34	0.980
S13—C53	1.724 (5)	C30—H35	0.980
S14—C54	1.715 (5)	C30—H36	0.980
S15—C55	1.718 (5)	C31—H37	0.980
S16—C56	1.725 (5)	C31—H38	0.980
P1—F1	1.612 (4)	C31—H39	0.980
P1—F2	1.586 (3)	C32—H40	0.980
P1—F3	1.591 (4)	C32—H41	0.980
P1—F4	1.598 (4)	C32—H42	0.980
P1—F5	1.598 (4)	C38—H43	0.980
P1—F6	1.590 (4)	C38—H44	0.980
P2—F7	1.578 (5)	C38—H45	0.980
P2—F8	1.591 (4)	C39—H46	0.980
P2—F9	1.602 (4)	C39—H47	0.980
P2—F10	1.588 (5)	C39—H48	0.980
P2—F11	1.557 (5)	C40—H49	0.980
P2—F12	1.593 (5)	C40—H50	0.980
O1—C25	1.203 (6)	C40—H51	0.980
O2—C26	1.206 (6)	C41—H52	0.980
O3—C25	1.354 (6)	C41—H53	0.980
O3—C29	1.459 (7)	C41—H54	0.980
O4—C26	1.346 (6)	C42—H55	0.980
O4—C30	1.457 (7)	C42—H56	0.980
O5—C27	1.198 (7)	C42—H57	0.980
O6—C28	1.200 (7)	C48—H58	0.980
O7—C27	1.347 (7)	C48—H59	0.980
O7—C31	1.466 (8)	C48—H60	0.980
O8—C28	1.334 (7)	C49—H61	0.980
O8—C32	1.458 (8)	C49—H62	0.980
O9—C57	1.199 (7)	C49—H63	0.980
O10—C58	1.204 (7)	C50—H64	0.980
O11—C57	1.339 (7)	C50—H65	0.980
O11—C61	1.451 (7)	C50—H66	0.980
O12—C58	1.333 (6)	C51—H67	0.980
O12—C62	1.453 (7)	C51—H68	0.980
O13—C59	1.201 (6)	C51—H69	0.980
O14—C60	1.205 (6)	C52—H70	0.980
O15—C59	1.347 (6)	C52—H71	0.980
O15—C63	1.452 (8)	C52—H72	0.980
O16—C60	1.340 (6)	C61—H73	0.980
O16—C64	1.457 (6)	C61—H74	0.980
C1—C2	1.436 (8)	C61—H75	0.980
C1—C5	1.418 (8)	C62—H76	0.980
C1—C6	1.501 (8)	C62—H77	0.980
C2—C3	1.420 (7)	C62—H78	0.980
C2—C7	1.492 (8)	C63—H79	0.980
C3—C4	1.445 (8)	C63—H80	0.980
C3—C8	1.492 (8)	C63—H81	0.980
C4—C5	1.423 (7)	C64—H82	0.980
C4—C9	1.495 (7)	C64—H83	0.980
C5—C10	1.495 (10)	C64—H84	0.980
C11—C12	1.420 (6)	Fe1—Fe2	3.1989 (10)
C11—C15	1.440 (8)	Fe1—Fe3	2.7418 (10)
C11—C16	1.492 (7)	Fe1—Fe4	2.7306 (10)
C12—C13	1.440 (7)	Fe2—Fe3	2.7505 (9)
C12—C17	1.504 (8)	Fe2—Fe4	2.7601 (11)
C13—C14	1.432 (7)	Fe3—Fe4	3.2499 (11)
C13—C18	1.487 (7)	Fe5—Fe6	3.1960 (10)
C14—C15	1.416 (7)	Fe5—Fe7	2.7603 (9)
C14—C19	1.480 (8)	Fe5—Fe8	2.7619 (11)
C15—C20	1.498 (7)	Fe6—Fe7	2.7601 (10)
C21—C22	1.363 (7)	Fe6—Fe8	2.7223 (10)
C21—C25	1.489 (8)	Fe7—Fe8	3.2216 (11)
			
Fe3—Fe1—Fe4	72.86 (2)	Fe1—C1—C5	69.7 (3)
Fe3—Fe1—S1	53.16 (4)	Fe1—C1—C6	129.3 (3)
Fe3—Fe1—S3	49.57 (3)	C2—C1—C5	107.5 (4)
Fe3—Fe1—S4	96.58 (4)	C2—C1—C6	124.4 (5)
Fe3—Fe1—C1	109.63 (15)	C5—C1—C6	127.7 (5)
Fe3—Fe1—C2	135.59 (13)	Fe1—C2—C1	70.6 (3)
Fe3—Fe1—C3	173.56 (14)	Fe1—C2—C3	69.6 (3)
Fe3—Fe1—C4	141.97 (17)	Fe1—C2—C7	133.0 (3)
Fe3—Fe1—C5	112.46 (16)	C1—C2—C3	108.7 (4)
Fe4—Fe1—S1	53.02 (3)	C1—C2—C7	123.0 (4)
Fe4—Fe1—S3	96.94 (4)	C3—C2—C7	127.7 (5)
Fe4—Fe1—S4	49.52 (3)	Fe1—C3—C2	72.4 (3)
Fe4—Fe1—C1	138.76 (16)	Fe1—C3—C4	68.8 (3)
Fe4—Fe1—C2	110.68 (15)	Fe1—C3—C8	128.6 (3)
Fe4—Fe1—C3	109.38 (15)	C2—C3—C4	107.2 (4)
Fe4—Fe1—C4	137.71 (16)	C2—C3—C8	126.9 (5)
Fe4—Fe1—C5	174.13 (17)	C4—C3—C8	125.7 (4)
S1—Fe1—S3	102.34 (5)	Fe1—C4—C3	71.7 (2)
S1—Fe1—S4	102.13 (5)	Fe1—C4—C5	71.9 (2)
S1—Fe1—C1	94.23 (14)	Fe1—C4—C9	128.3 (4)
S1—Fe1—C2	92.28 (13)	C3—C4—C5	107.9 (4)
S1—Fe1—C3	123.00 (15)	C3—C4—C9	124.4 (5)
S1—Fe1—C4	156.35 (16)	C5—C4—C9	127.3 (5)
S1—Fe1—C5	127.68 (14)	Fe1—C5—C1	72.2 (3)
S3—Fe1—S4	80.40 (5)	Fe1—C5—C4	69.2 (3)
S3—Fe1—C1	116.13 (17)	Fe1—C5—C10	128.6 (4)
S3—Fe1—C2	152.20 (15)	C1—C5—C4	108.7 (5)
S3—Fe1—C3	134.66 (14)	C1—C5—C10	126.2 (5)
S3—Fe1—C4	97.07 (17)	C4—C5—C10	124.9 (5)
S3—Fe1—C5	88.60 (17)	Fe2—C11—C12	69.2 (3)
S4—Fe1—C1	153.79 (16)	Fe2—C11—C15	70.2 (3)
S4—Fe1—C2	119.78 (15)	Fe2—C11—C16	133.2 (3)
S4—Fe1—C3	89.28 (15)	C12—C11—C15	107.4 (4)
S4—Fe1—C4	94.26 (15)	C12—C11—C16	126.2 (5)
S4—Fe1—C5	130.18 (14)	C15—C11—C16	125.7 (4)
C1—Fe1—C2	38.3 (2)	Fe2—C12—C11	72.6 (3)
C1—Fe1—C3	64.6 (2)	Fe2—C12—C13	69.8 (3)
C1—Fe1—C4	64.8 (2)	Fe2—C12—C17	128.9 (3)
C1—Fe1—C5	38.1 (2)	C11—C12—C13	108.3 (4)
C2—Fe1—C3	38.07 (19)	C11—C12—C17	125.8 (5)
C2—Fe1—C4	64.6 (2)	C13—C12—C17	125.5 (4)
C2—Fe1—C5	63.9 (2)	Fe2—C13—C12	70.9 (2)
C3—Fe1—C4	39.5 (2)	Fe2—C13—C14	70.9 (2)
C3—Fe1—C5	65.1 (2)	Fe2—C13—C18	127.5 (3)
C4—Fe1—C5	38.9 (2)	C12—C13—C14	107.6 (4)
Fe3—Fe2—Fe4	72.28 (2)	C12—C13—C18	125.8 (4)
Fe3—Fe2—S2	52.91 (3)	C14—C13—C18	126.4 (5)
Fe3—Fe2—S3	49.43 (3)	Fe2—C14—C13	70.0 (2)
Fe3—Fe2—S4	96.47 (4)	Fe2—C14—C15	72.2 (2)
Fe3—Fe2—C11	109.18 (13)	Fe2—C14—C19	128.9 (4)
Fe3—Fe2—C12	115.51 (14)	C13—C14—C15	108.0 (5)
Fe3—Fe2—C13	147.54 (15)	C13—C14—C19	126.2 (4)
Fe3—Fe2—C14	169.73 (14)	C15—C14—C19	125.5 (4)
Fe3—Fe2—C15	131.92 (14)	Fe2—C15—C11	71.3 (2)
Fe4—Fe2—S2	53.25 (4)	Fe2—C15—C14	69.6 (2)
Fe4—Fe2—S3	96.12 (4)	Fe2—C15—C20	130.1 (3)
Fe4—Fe2—S4	49.08 (3)	C11—C15—C14	108.7 (4)
Fe4—Fe2—C11	142.57 (14)	C11—C15—C20	124.5 (5)
Fe4—Fe2—C12	172.11 (14)	C14—C15—C20	126.6 (5)
Fe4—Fe2—C13	133.12 (16)	S5—C21—C22	118.6 (4)
Fe4—Fe2—C14	107.23 (16)	S5—C21—C25	116.1 (3)
Fe4—Fe2—C15	111.85 (16)	C22—C21—C25	125.3 (4)
S2—Fe2—S3	101.98 (5)	S6—C22—C21	120.0 (4)
S2—Fe2—S4	102.01 (5)	S6—C22—C26	115.6 (3)
S2—Fe2—C11	96.54 (13)	C21—C22—C26	124.3 (5)
S2—Fe2—C12	131.81 (14)	S7—C23—C24	119.1 (4)
S2—Fe2—C13	154.35 (14)	S7—C23—C27	117.0 (4)
S2—Fe2—C14	118.21 (14)	C24—C23—C27	123.9 (5)
S2—Fe2—C15	89.83 (13)	S8—C24—C23	119.4 (4)
S3—Fe2—S4	80.53 (5)	S8—C24—C28	116.4 (4)
S3—Fe2—C11	113.25 (15)	C23—C24—C28	124.2 (5)
S3—Fe2—C12	88.68 (14)	O1—C25—O3	124.1 (5)
S3—Fe2—C13	101.65 (14)	O1—C25—C21	124.4 (4)
S3—Fe2—C14	139.81 (14)	O3—C25—C21	111.6 (4)
S3—Fe2—C15	151.14 (17)	O2—C26—O4	124.6 (4)
S4—Fe2—C11	154.08 (13)	O2—C26—C22	123.5 (4)
S4—Fe2—C12	126.15 (14)	O4—C26—C22	111.9 (4)
S4—Fe2—C13	91.59 (15)	O5—C27—O7	124.6 (5)
S4—Fe2—C14	90.46 (16)	O5—C27—C23	124.8 (5)
S4—Fe2—C15	123.03 (16)	O7—C27—C23	110.6 (4)
C11—Fe2—C12	38.16 (18)	O6—C28—O8	125.2 (6)
C11—Fe2—C13	64.78 (19)	O6—C28—C24	123.6 (5)
C11—Fe2—C14	64.7 (2)	O8—C28—C24	111.2 (4)
C11—Fe2—C15	38.5 (2)	Fe5—C33—C34	69.2 (3)
C12—Fe2—C13	39.3 (2)	Fe5—C33—C37	70.1 (3)
C12—Fe2—C14	65.4 (2)	Fe5—C33—C38	133.0 (3)
C12—Fe2—C15	64.4 (2)	C34—C33—C37	107.1 (4)
C13—Fe2—C14	39.1 (2)	C34—C33—C38	128.1 (5)
C13—Fe2—C15	64.56 (18)	C37—C33—C38	124.2 (4)
C14—Fe2—C15	38.2 (2)	Fe5—C34—C33	72.7 (3)
Fe1—Fe3—Fe2	71.24 (2)	Fe5—C34—C35	68.9 (3)
Fe1—Fe3—S1	50.68 (3)	Fe5—C34—C39	127.1 (4)
Fe1—Fe3—S2	95.66 (4)	C33—C34—C35	108.8 (4)
Fe1—Fe3—S3	52.77 (4)	C33—C34—C39	124.4 (4)
Fe1—Fe3—S5	160.90 (4)	C35—C34—C39	126.7 (4)
Fe1—Fe3—S6	97.96 (4)	Fe5—C35—C34	72.1 (2)
Fe2—Fe3—S1	95.97 (4)	Fe5—C35—C36	71.3 (2)
Fe2—Fe3—S2	50.10 (3)	Fe5—C35—C40	125.2 (3)
Fe2—Fe3—S3	52.60 (3)	C34—C35—C36	107.7 (4)
Fe2—Fe3—S5	97.86 (4)	C34—C35—C40	126.7 (4)
Fe2—Fe3—S6	162.40 (4)	C36—C35—C40	125.5 (5)
S1—Fe3—S2	78.75 (5)	Fe5—C36—C35	68.8 (2)
S1—Fe3—S3	103.06 (5)	Fe5—C36—C37	72.4 (2)
S1—Fe3—S5	148.17 (5)	Fe5—C36—C41	129.2 (4)
S1—Fe3—S6	87.00 (5)	C35—C36—C37	107.3 (4)
S2—Fe3—S3	102.34 (5)	C35—C36—C41	126.2 (4)
S2—Fe3—S5	88.46 (5)	C37—C36—C41	126.2 (4)
S2—Fe3—S6	147.04 (5)	Fe5—C37—C33	71.4 (3)
S3—Fe3—S5	108.13 (6)	Fe5—C37—C36	69.6 (2)
S3—Fe3—S6	109.82 (5)	Fe5—C37—C42	129.0 (3)
S5—Fe3—S6	88.34 (5)	C33—C37—C36	109.2 (4)
Fe1—Fe4—Fe2	71.26 (2)	C33—C37—C42	124.2 (4)
Fe1—Fe4—S1	50.93 (3)	C36—C37—C42	126.4 (5)
Fe1—Fe4—S2	95.53 (4)	Fe6—C43—C44	70.0 (3)
Fe1—Fe4—S4	53.14 (3)	Fe6—C43—C47	70.0 (3)
Fe1—Fe4—S7	166.99 (4)	Fe6—C43—C48	132.6 (3)
Fe1—Fe4—S8	94.25 (4)	C44—C43—C47	109.2 (4)
Fe2—Fe4—S1	95.99 (4)	C44—C43—C48	126.0 (5)
Fe2—Fe4—S2	49.84 (3)	C47—C43—C48	124.2 (4)
Fe2—Fe4—S4	52.45 (4)	Fe6—C44—C43	72.0 (3)
Fe2—Fe4—S7	101.91 (5)	Fe6—C44—C45	68.8 (2)
Fe2—Fe4—S8	156.96 (4)	Fe6—C44—C49	129.7 (3)
S1—Fe4—S2	78.61 (5)	C43—C44—C45	106.7 (4)
S1—Fe4—S4	103.64 (5)	C43—C44—C49	127.1 (5)
S1—Fe4—S7	142.01 (5)	C45—C44—C49	125.8 (4)
S1—Fe4—S8	87.79 (5)	Fe6—C45—C44	71.8 (2)
S2—Fe4—S4	101.96 (5)	Fe6—C45—C46	71.6 (2)
S2—Fe4—S7	87.78 (5)	Fe6—C45—C50	128.4 (4)
S2—Fe4—S8	152.40 (5)	C44—C45—C46	109.0 (4)
S4—Fe4—S7	113.87 (5)	C44—C45—C50	124.4 (4)
S4—Fe4—S8	104.56 (6)	C46—C45—C50	126.2 (5)
S7—Fe4—S8	88.46 (6)	Fe6—C46—C45	69.5 (2)
Fe7—Fe5—Fe8	71.38 (2)	Fe6—C46—C47	71.4 (3)
Fe7—Fe5—S9	52.56 (3)	Fe6—C46—C51	128.3 (4)
Fe7—Fe5—S11	49.28 (3)	C45—C46—C47	107.6 (5)
Fe7—Fe5—S12	96.31 (4)	C45—C46—C51	127.2 (5)
Fe7—Fe5—C33	110.00 (14)	C47—C46—C51	125.0 (4)
Fe7—Fe5—C34	114.40 (15)	Fe6—C47—C43	71.4 (2)
Fe7—Fe5—C35	144.57 (15)	Fe6—C47—C46	69.9 (2)
Fe7—Fe5—C36	172.18 (13)	Fe6—C47—C52	131.4 (3)
Fe7—Fe5—C37	134.36 (13)	C43—C47—C46	107.5 (4)
Fe8—Fe5—S9	53.02 (4)	C43—C47—C52	124.8 (5)
Fe8—Fe5—S11	95.46 (4)	C46—C47—C52	127.1 (5)
Fe8—Fe5—S12	48.94 (3)	S13—C53—C54	118.9 (4)
Fe8—Fe5—C33	141.45 (14)	S13—C53—C57	118.7 (4)
Fe8—Fe5—C34	174.23 (15)	C54—C53—C57	122.1 (5)
Fe8—Fe5—C35	135.87 (16)	S14—C54—C53	119.0 (4)
Fe8—Fe5—C36	108.88 (16)	S14—C54—C58	115.7 (3)
Fe8—Fe5—C37	112.17 (15)	C53—C54—C58	125.2 (4)
S9—Fe5—S11	101.51 (5)	S15—C55—C56	118.9 (4)
S9—Fe5—S12	101.71 (5)	S15—C55—C59	115.9 (3)
S9—Fe5—C33	95.87 (13)	C56—C55—C59	125.1 (4)
S9—Fe5—C34	130.06 (14)	S16—C56—C55	119.6 (4)
S9—Fe5—C35	156.50 (14)	S16—C56—C60	115.4 (4)
S9—Fe5—C36	121.00 (13)	C55—C56—C60	125.0 (5)
S9—Fe5—C37	91.73 (13)	O9—C57—O11	124.2 (4)
S11—Fe5—S12	80.94 (5)	O9—C57—C53	123.9 (5)
S11—Fe5—C33	114.74 (15)	O11—C57—C53	111.9 (4)
S11—Fe5—C34	88.67 (15)	O10—C58—O12	124.7 (5)
S11—Fe5—C35	99.01 (14)	O10—C58—C54	123.4 (4)
S11—Fe5—C36	137.49 (13)	O12—C58—C54	111.9 (4)
S11—Fe5—C37	151.92 (16)	O13—C59—O15	125.2 (5)
S12—Fe5—C33	153.63 (14)	O13—C59—C55	124.1 (5)
S12—Fe5—C34	128.22 (14)	O15—C59—C55	110.7 (4)
S12—Fe5—C35	92.68 (16)	O14—C60—O16	125.2 (4)
S12—Fe5—C36	89.33 (15)	O14—C60—C56	124.3 (4)
S12—Fe5—C37	120.75 (15)	O16—C60—C56	110.3 (4)
C33—Fe5—C34	38.08 (19)	C1—C6—H1	109.5
C33—Fe5—C35	64.9 (2)	C1—C6—H2	109.5
C33—Fe5—C36	64.7 (2)	C1—C6—H3	109.5
C33—Fe5—C37	38.5 (2)	H1—C6—H2	109.5
C34—Fe5—C35	39.0 (2)	H1—C6—H3	109.5
C34—Fe5—C36	65.4 (2)	H2—C6—H3	109.5
C34—Fe5—C37	64.2 (2)	C2—C7—H4	109.5
C35—Fe5—C36	39.9 (2)	C2—C7—H5	109.5
C35—Fe5—C37	64.86 (19)	C2—C7—H6	109.5
C36—Fe5—C37	37.96 (18)	H4—C7—H5	109.5
Fe7—Fe6—Fe8	71.97 (2)	H4—C7—H6	109.5
Fe7—Fe6—S10	52.76 (4)	H5—C7—H6	109.5
Fe7—Fe6—S11	49.31 (3)	C3—C8—H7	109.5
Fe7—Fe6—S12	96.31 (4)	C3—C8—H8	109.5
Fe7—Fe6—C43	137.19 (13)	C3—C8—H9	109.5
Fe7—Fe6—C44	174.94 (14)	H7—C8—H8	109.5
Fe7—Fe6—C45	141.21 (16)	H7—C8—H9	109.5
Fe7—Fe6—C46	111.99 (15)	H8—C8—H9	109.5
Fe7—Fe6—C47	110.24 (14)	C4—C9—H10	109.5
Fe8—Fe6—S10	53.06 (3)	C4—C9—H11	109.5
Fe8—Fe6—S11	96.70 (4)	C4—C9—H12	109.5
Fe8—Fe6—S12	49.57 (3)	H10—C9—H11	109.5
Fe8—Fe6—C43	110.93 (14)	H10—C9—H12	109.5
Fe8—Fe6—C44	110.25 (15)	H11—C9—H12	109.5
Fe8—Fe6—C45	138.98 (15)	C5—C10—H13	109.5
Fe8—Fe6—C46	175.26 (17)	C5—C10—H14	109.5
Fe8—Fe6—C47	138.22 (15)	C5—C10—H15	109.5
S10—Fe6—S11	101.81 (5)	H13—C10—H14	109.5
S10—Fe6—S12	102.28 (5)	H13—C10—H15	109.5
S10—Fe6—C43	93.42 (13)	H14—C10—H15	109.5
S10—Fe6—C44	124.58 (15)	C11—C16—H16	109.5
S10—Fe6—C45	156.70 (16)	C11—C16—H17	109.5
S10—Fe6—C46	126.85 (14)	C11—C16—H18	109.5
S10—Fe6—C47	93.71 (13)	H16—C16—H17	109.5
S11—Fe6—S12	81.05 (5)	H16—C16—H18	109.5
S11—Fe6—C43	152.28 (15)	H17—C16—H18	109.5
S11—Fe6—C44	133.61 (15)	C12—C17—H19	109.5
S11—Fe6—C45	96.38 (16)	C12—C17—H20	109.5
S11—Fe6—C46	87.96 (17)	C12—C17—H21	109.5
S11—Fe6—C47	116.51 (16)	H19—C17—H20	109.5
S12—Fe6—C43	118.38 (14)	H19—C17—H21	109.5
S12—Fe6—C44	88.47 (14)	H20—C17—H21	109.5
S12—Fe6—C45	94.77 (15)	C13—C18—H22	109.5
S12—Fe6—C46	130.87 (14)	C13—C18—H23	109.5
S12—Fe6—C47	153.44 (15)	C13—C18—H24	109.5
C43—Fe6—C44	37.94 (19)	H22—C18—H23	109.5
C43—Fe6—C45	64.3 (2)	H22—C18—H24	109.5
C43—Fe6—C46	64.4 (2)	H23—C18—H24	109.5
C43—Fe6—C47	38.6 (2)	C14—C19—H25	109.5
C44—Fe6—C45	39.4 (2)	C14—C19—H26	109.5
C44—Fe6—C46	65.6 (2)	C14—C19—H27	109.5
C44—Fe6—C47	64.97 (19)	H25—C19—H26	109.5
C45—Fe6—C46	39.0 (2)	H25—C19—H27	109.5
C45—Fe6—C47	64.92 (19)	H26—C19—H27	109.5
C46—Fe6—C47	38.7 (2)	C15—C20—H28	109.5
Fe5—Fe7—Fe6	70.75 (2)	C15—C20—H29	109.5
Fe5—Fe7—S9	50.18 (3)	C15—C20—H30	109.5
Fe5—Fe7—S10	96.41 (4)	H28—C20—H29	109.5
Fe5—Fe7—S11	52.42 (3)	H28—C20—H30	109.5
Fe5—Fe7—S13	167.60 (5)	H29—C20—H30	109.5
Fe5—Fe7—S14	94.49 (4)	O3—C29—H31	109.5
Fe6—Fe7—S9	95.81 (4)	O3—C29—H32	109.5
Fe6—Fe7—S10	50.43 (3)	O3—C29—H33	109.5
Fe6—Fe7—S11	52.28 (4)	H31—C29—H32	109.5
Fe6—Fe7—S13	102.21 (4)	H31—C29—H33	109.5
Fe6—Fe7—S14	155.98 (5)	H32—C29—H33	109.4
S9—Fe7—S10	80.11 (5)	O4—C30—H34	109.5
S9—Fe7—S11	102.27 (5)	O4—C30—H35	109.5
S9—Fe7—S13	142.09 (5)	O4—C30—H36	109.5
S9—Fe7—S14	88.23 (5)	H34—C30—H35	109.5
S10—Fe7—S11	102.45 (5)	H34—C30—H36	109.5
S10—Fe7—S13	86.10 (5)	H35—C30—H36	109.5
S10—Fe7—S14	153.08 (5)	O7—C31—H37	109.5
S11—Fe7—S13	115.18 (5)	O7—C31—H38	109.5
S11—Fe7—S14	103.71 (6)	O7—C31—H39	109.5
S13—Fe7—S14	88.50 (6)	H37—C31—H38	109.5
Fe5—Fe8—Fe6	71.29 (2)	H37—C31—H39	109.5
Fe5—Fe8—S9	50.05 (3)	H38—C31—H39	109.5
Fe5—Fe8—S10	96.67 (4)	O8—C32—H40	109.5
Fe5—Fe8—S12	52.31 (4)	O8—C32—H41	109.5
Fe5—Fe8—S15	156.34 (4)	O8—C32—H42	109.5
Fe5—Fe8—S16	100.19 (5)	H40—C32—H41	109.5
Fe6—Fe8—S9	96.43 (4)	H40—C32—H42	109.5
Fe6—Fe8—S10	51.11 (3)	H41—C32—H42	109.5
Fe6—Fe8—S12	53.04 (3)	C33—C38—H43	109.5
Fe6—Fe8—S15	94.10 (4)	C33—C38—H44	109.5
Fe6—Fe8—S16	163.16 (4)	C33—C38—H45	109.5
S9—Fe8—S10	80.00 (5)	H43—C38—H44	109.5
S9—Fe8—S12	102.11 (5)	H43—C38—H45	109.5
S9—Fe8—S15	153.04 (5)	H44—C38—H45	109.5
S9—Fe8—S16	88.30 (5)	C34—C39—H46	109.5
S10—Fe8—S12	103.78 (5)	C34—C39—H47	109.5
S10—Fe8—S15	87.56 (5)	C34—C39—H48	109.5
S10—Fe8—S16	145.71 (5)	H46—C39—H47	109.5
S12—Fe8—S15	104.05 (5)	H46—C39—H48	109.5
S12—Fe8—S16	110.18 (5)	H47—C39—H48	109.5
S15—Fe8—S16	88.75 (5)	C35—C40—H49	109.5
Fe1—S1—Fe3	76.17 (4)	C35—C40—H50	109.5
Fe1—S1—Fe4	76.05 (4)	C35—C40—H51	109.5
Fe3—S1—Fe4	92.27 (5)	H49—C40—H50	109.5
Fe2—S2—Fe3	76.99 (4)	H49—C40—H51	109.5
Fe2—S2—Fe4	76.91 (4)	H50—C40—H51	109.5
Fe3—S2—Fe4	91.88 (5)	C36—C41—H52	109.5
Fe1—S3—Fe2	91.42 (5)	C36—C41—H53	109.5
Fe1—S3—Fe3	77.67 (5)	C36—C41—H54	109.5
Fe2—S3—Fe3	77.97 (4)	H52—C41—H53	109.5
Fe1—S4—Fe2	91.33 (5)	H52—C41—H54	109.5
Fe1—S4—Fe4	77.34 (4)	H53—C41—H54	109.5
Fe2—S4—Fe4	78.47 (5)	C37—C42—H55	109.5
Fe3—S5—C21	106.63 (19)	C37—C42—H56	109.5
Fe3—S6—C22	106.32 (18)	C37—C42—H57	109.5
Fe4—S7—C23	106.26 (19)	H55—C42—H56	109.5
Fe4—S8—C24	106.5 (2)	H55—C42—H57	109.5
Fe5—S9—Fe7	77.27 (4)	H56—C42—H57	109.5
Fe5—S9—Fe8	76.93 (4)	C43—C48—H58	109.5
Fe7—S9—Fe8	91.13 (5)	C43—C48—H59	109.5
Fe6—S10—Fe7	76.80 (5)	C43—C48—H60	109.5
Fe6—S10—Fe8	75.83 (4)	H58—C48—H59	109.5
Fe7—S10—Fe8	91.40 (5)	H58—C48—H60	109.5
Fe5—S11—Fe6	91.48 (5)	H59—C48—H60	109.5
Fe5—S11—Fe7	78.30 (4)	C44—C49—H61	109.5
Fe6—S11—Fe7	78.41 (5)	C44—C49—H62	109.5
Fe5—S12—Fe6	91.62 (5)	C44—C49—H63	109.5
Fe5—S12—Fe8	78.75 (5)	H61—C49—H62	109.5
Fe6—S12—Fe8	77.40 (4)	H61—C49—H63	109.5
Fe7—S13—C53	106.56 (18)	H62—C49—H63	109.5
Fe7—S14—C54	106.8 (2)	C45—C50—H64	109.5
Fe8—S15—C55	106.3 (2)	C45—C50—H65	109.5
Fe8—S16—C56	105.81 (18)	C45—C50—H66	109.5
F1—P1—F2	179.4 (2)	H64—C50—H65	109.5
F1—P1—F3	90.2 (2)	H64—C50—H66	109.5
F1—P1—F4	89.1 (2)	H65—C50—H66	109.5
F1—P1—F5	89.4 (2)	C46—C51—H67	109.5
F1—P1—F6	89.1 (2)	C46—C51—H68	109.5
F2—P1—F3	90.1 (2)	C46—C51—H69	109.5
F2—P1—F4	91.5 (2)	H67—C51—H68	109.5
F2—P1—F5	90.4 (2)	H67—C51—H69	109.5
F2—P1—F6	90.3 (2)	H68—C51—H69	109.5
F3—P1—F4	90.3 (2)	C47—C52—H70	109.5
F3—P1—F5	179.1 (2)	C47—C52—H71	109.5
F3—P1—F6	89.8 (2)	C47—C52—H72	109.5
F4—P1—F5	90.4 (2)	H70—C52—H71	109.5
F4—P1—F6	178.2 (2)	H70—C52—H72	109.5
F5—P1—F6	89.5 (2)	H71—C52—H72	109.5
F7—P2—F8	175.7 (3)	O11—C61—H73	109.5
F7—P2—F9	88.7 (3)	O11—C61—H74	109.5
F7—P2—F10	88.1 (3)	O11—C61—H75	109.5
F7—P2—F11	92.8 (4)	H73—C61—H74	109.5
F7—P2—F12	91.9 (3)	H73—C61—H75	109.5
F8—P2—F9	87.1 (3)	H74—C61—H75	109.5
F8—P2—F10	90.7 (2)	O12—C62—H76	109.5
F8—P2—F11	91.3 (3)	O12—C62—H77	109.5
F8—P2—F12	89.2 (2)	O12—C62—H78	109.5
F9—P2—F10	88.9 (2)	H76—C62—H77	109.5
F9—P2—F11	177.7 (4)	H76—C62—H78	109.5
F9—P2—F12	90.1 (2)	H77—C62—H78	109.5
F10—P2—F11	89.4 (3)	O15—C63—H79	109.5
F10—P2—F12	179.0 (2)	O15—C63—H80	109.5
F11—P2—F12	91.6 (3)	O15—C63—H81	109.5
C25—O3—C29	115.0 (4)	H79—C63—H80	109.5
C26—O4—C30	114.2 (4)	H79—C63—H81	109.5
C27—O7—C31	115.1 (4)	H80—C63—H81	109.5
C28—O8—C32	114.2 (4)	O16—C64—H82	109.5
C57—O11—C61	114.5 (5)	O16—C64—H83	109.5
C58—O12—C62	116.0 (4)	O16—C64—H84	109.5
C59—O15—C63	114.5 (4)	H82—C64—H83	109.5
C60—O16—C64	114.8 (3)	H82—C64—H84	109.5
Fe1—C1—C2	71.2 (3)	H83—C64—H84	109.5
